# Updates to Article Categories for Prehospital and Disaster Medicine

**DOI:** 10.1017/S1049023X24000633

**Published:** 2024-10

**Authors:** Jeffrey Michael Franc

**Affiliations:** 1. Research Director, Department of Emergency Medicine, University of Alberta, Edmonton, Alberta, Canada; 2. Associate Professor, Faculty of Medicine, University of Alberta, Edmonton, Alberta, Canada; 3. Visiting Professor in Disaster Medicine, Università del Piemonte Orientale, Italy

**Keywords:** article categories, publication, research

## Abstract

For 2025, *Prehospital and Disaster Medicine* will be updating the available article categories. These changes assure that article categories are better aligned with the recently updated *Prehospital and Disaster Medicine* mission statement. The updated article categories will facilitate the publication of innovative, high-impact, evidence-based research in both prehospital and Disaster Medicine.

For 2025, *Prehospital and Disaster Medicine* will be updating the available article categories. This includes changes to the existing article categories and the addition of several new options (Table 1). These changes assure that article categories are better aligned with the recently updated *Prehospital and Disaster Medicine* mission statement, which was featured in the August 2024 issue.^1^ The updated article categories will facilitate the publication of innovative, high-impact, evidence-based research in both prehospital and Disaster Medicine.

**Table 1.** Article Categories

Previous Article Category Name	New Article Category Name
Original Research	Unchanged
Systematic Review	Structured Review
Research Report	Innovation Report
Field Report	Disaster Report
Commentary	Unchanged
Guest Editorial	Unchanged

## Article Categories

### Original Research

The Original Research category will remain largely unchanged. Manuscripts published as original research are structured research that uses quantitative or qualitative data collection methods and analyses to establish a hypothesis, association, or prove a cause-and-effect relationship. Manuscripts are expected to be innovative, high-impact, and evidence based. In addition, they should articulate a strong value proposition and have external validity. Sampling technique and sample size are expected to be free of bias. Randomized controlled trials should adhere to the CONSORT Statement.^2^ The CONSORT checklist must be submitted as supplemental material when a randomized trial is submitted for consideration. Diagnostic studies should follow the STARD Guidelines.^3^

The abstract must be limited to 375 words and include the subheadings of Introduction, Study Objective, Methods, Results, and Conclusions.

The main manuscript text cannot exceed 4,000 words. A concise statement of the study hypothesis or objective must be included in the last paragraph of the introduction section. The methods should include the statistical analysis used for each of the study objectives. A statement of ethical approval must appear in the methods section. Limitations of the study must be discussed, preferably as a separate subsection, at the end of the discussion section. The conclusions should present the value proposition concisely without subjective statements, editorialization, or statements that extend beyond the findings of the study.

Original Research manuscript must be formatted as follows:

1. Title page (this page is not made available to peer reviewers of a submission);
2. List of abbreviations and symbols used and the meaning of each;
3. Structured abstract;
4. Manuscript body with the following headings:
  - **Introduction**
  - **Methods**
  - **Results**
  - **Discussion**
  - **Limitations**
  - **Conclusions**
5. References (a numbered list of references in the order in which they appear in the text);
6. Tables/Charts/Figures in order referenced in main text;
7. Units of measure list or statement (when applicable).

### Structured Reviews

This new format replaces the previous category of Systematic Reviews. These are structured, rigorous reviews of published and “grey” literature to be used to clarify areas in which there is lack of consensus. This may include meta-analysis, systematic review, realist review, or scoping review. The format for submission should be the same as that described above for Original Research. Main text should not exceed 4,000 words (abstract 375 words). Reviews must adhere to the PRISMA method^4^ and the appropriate PRISMA Guidelines must be attached as supplemental material. The last paragraph of the introduction should clearly state the review question.

In all cases, reviews should provide a clear value-proposition. Reviews that simply summarize what studies are available, or whose major conclusion is that insufficient evidence is available, are strongly discouraged. Narrative and unstructured reviews are not suitable for submission to *Prehospital and Disaster Medicine*.

### Innovation Report

*Prehospital and Disaster Medicine* encourages the early publication of innovative concepts. This new format replaces the previous category of Research Report. An innovation report is a structured report that describes preliminary research findings, activities, or aspects of science that provide information for the progression of knowledge or understanding in the areas of focus for the journal. Innovation reports must describe a novel concept with a clear value proposition. This includes pilot studies, initial reports of innovations, and proof of concept papers. The abstract and manuscript should be formatted as detailed above for Original Research. The manuscript should not exceed 2,000 words (not including the abstract).

### Disaster Report

Previously called Field Reports, Disaster Reports provide an analysis of prehospital, emergency health, or disaster events. The main text of a Disaster Report submitted to *Prehospital and Disaster Medicine* should be no more than 2,000 words in length in addition to a 250-word unstructured abstract. Supporting maps, graphs, and tables are encouraged.

Disaster Report format must include the following sections:

1. **Event Description:** Including a list of specific event identifiers:
  - **Event type:** (example: tropical storm, bombing, train crash, mass-gathering event);
  - **Event Date;**
  - **Location:** Include geographic coordinates in latitude, longitude, elevation where necessary;
  - **Response type:** (example: medical relief, humanitarian, public health surveillance);
2. **Abstract:** A 250-word unstructured summary;
3. **Introduction:** A summary of the event with specific data available, such as population density, detailed event description, general damage that occurred, and the author’s role in the response;
4. **Source:** The source of information and data used for the report, including the name of the health ethics review board providing approval and the reference number;
5. **Observations:** A detailed description of observations made;
6. **Analysis:** Details and interpretation of any quantitative or qualitative analysis;
7. **References**: A numbered list of references in the order in which they appear in the text.

### Commentary

Article commentaries expand on the methods, findings, or interpretation of a manuscript previously published in *Prehospital and Disaster Medicine*. Commentaries must cite the original manuscript. Political or religious statements and criticisms based upon individual opinion are not acceptable. Studies that include new data should not be submitted to this category. Self-citation and data churning are discouraged. While authors may choose their format, submissions should be well focused with a maximum length of 1,500 words. A maximum of 15 references and two figures are allowed.

### Guest Editorials

Guest Editorial submissions are considered by invitation only. Please contact the *Prehospital and Disaster Medicine* Editor-in-Chief to discuss potential Guest Editorials before submission. Political, religious, biased criticisms, or advertisement material are not accepted.

## Title Page

In addition to updates to article categories, several updates to the title page format will be introduced. The title page must include the following sections:

1. **Title:** Include the study design in the title where possible;
2. **Authors**: First name, middle initials, last names, and highest academic degrees of all authors (abbreviated as MD, MPH, etc.), along with institutions each author is affiliated;
3. **Corresponding Author**: Contact details for the corresponding author;
4. **Conflict of Interest Statement:** List each author’s declaration individually;
5. **Ethical Approval:** Name of the institution granting ethical permission and the reference number;
6. **Author Contributions:** Describe each author’s contribution individually, as each author must meet all four of the ICMJE’s authorship criteria;^5^
7. **Use of AI Technology:** A statement detailing which, if any, Artificial Intelligence (AI) tools were used for constructing the manuscript;
8. **Keywords:** Three to five keywords or phrases in alphabetical order separated by semicolons to facilitate indexing or electronic searches using the US National Library of Medicine Medical Subject Headings database;^6^
9. **Abbreviations:** List of abbreviations and symbols used and the meaning of each;
10. **Word Count:** Include a separate word count for the abstract and manuscript.

## Summary

*Prehospital and Disaster Medicine*’s new article categories are designed to provide additional clarity to authors as well as additional options. Articles submitted in 2025 will be required to adhere to the new guidelines. Instructions for authors will also be updated for 2025 and will be the topic of the next issue’s editorial.
